# Supplementary material for: Implantable optical fibers for immunotherapeutics delivery and tumor impedance measurement
Source: Nat Commun. 2021 Aug 26;12:5138. doi: 10.1038/s41467-021-25391-z (PMC8390758; doi:10.1038/s41467-021-25391-z)
Supplement: Supplementary file 1 — Supplementary Information [file 41467_2021_25391_MOESM1_ESM.pdf]

Implantable Optical Fibers for Immunotherapeutics Delivery and Tumor Impedance Measurement

Ai Lin Chin,[1,*] Shan Jiang,[2,*] Eungyo Jang,[1] Liqian Niu,[1] Liwu Li,[3] Xiaoting Jia,[2] Rong Tong[1]

[1] Department of Chemical Engineering, Virginia Polytechnic Institute and State University, 635 Prices Fork Road, Blacksburg, Virginia, 24061, United States

[2] Bradley Department of Electrical and Computer Engineering, 1185 Perry Street, Virginia Polytechnic Institute and State University, Blacksburg, Virginia, 24061, United States

[3] Department of Biological Science, 970 Washington Street SW, Virginia Polytechnic Institute and State University, Blacksburg, Virginia, 24061, United States

Correspondence should be addressed to X.J. (E-mail: xjia@vt.edu) and R.T. (E-mail: rtong@vt.edu).

* These authors contributed equally to this work.

Supplementary Information

Table of Contents

1. Materials S-3

2. Supplementary Figures 1-19 S-4

3. Supplementary Table 1 S-27

4. Circuit Model Discussion S-28

5. References S-30

1. Materials

1.1 PLA synthesis

The PLA used for fiber coating was synthesized based on literature.[1] In brief, 10 g lactide (3,6-dimethyl-1,4-dioxane-2,5-dione, Sigma-Aldrich, St Louis, MO) and tin(II) 2-ethylhexanoate (0.01 equiv., Sigma-Aldrich) were added into a cylindrical heavy wall pressure vessel equipped with magnetic stirring bar and heated to 140 °C in an oil bath. Mixture was stirred and left to react for 6 hours. After the crude mixture had cooled to room temperature, it was added into methanol at 0°C. White thread-like PLA polymer was precipitated in cold methanol and pooled into a 50 mL polypropylene tube before completely dried under vacuum.

1.2 Cy7-BSA synthesis.

50 mg BSA (Sigma-Aldrich) was dissolved in 5 mL PBS in a 20 mL reaction vial. 5 mg Cy7-NHS ester (Lumiprobe) and *N*-(3-Dimethylaminopropyl)-*N'*-ethylcarbodiimide hydrochloride (EDC, 4 equiv., Sigma-Aldrich) were dissolved in 0.5 mL PBS and immediately transferred to the dissolved BSA solution. The crude mixture was agitated vigorously and left to react overnight in dark. The resulting mixture was then dialyzed and lyophilized to obtain dried blue powder that was stored at -80 °C.

2. Supplementary Figures

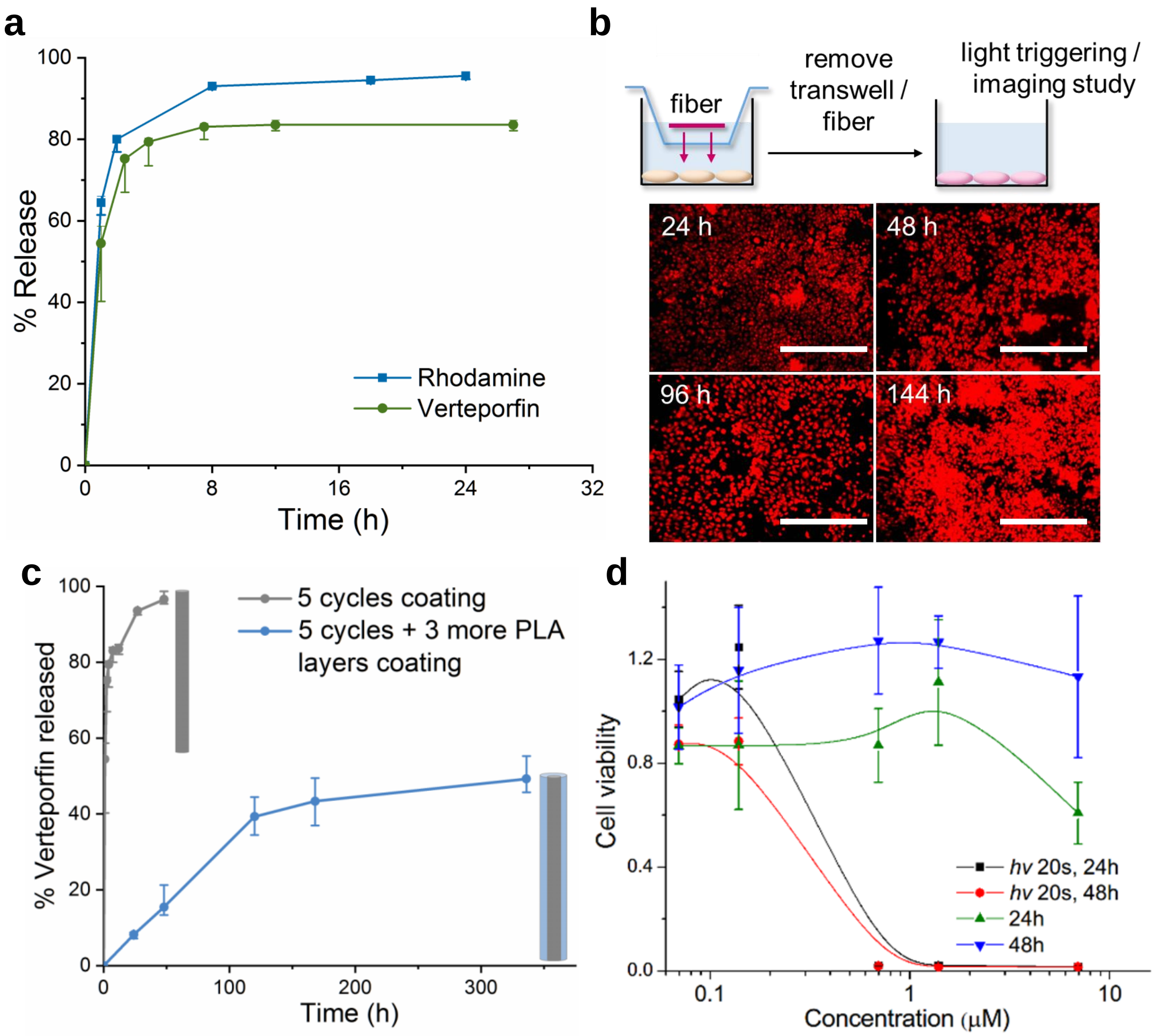

Supplementary Figure 1. *In vitro* sustained release of drugs loaded onto the fiber in IMOD. **a**, Cumulative release of verteporfin or rhodamine over time (*n* = 5). Data are medians ± quartiles. **b**, Schematic of transwell assays for *in vitro* imaging and toxicity studies. Fluorescent images were taken of 4T1 cells incubated with rhodamine loaded fibers in transwell between 24 to 144 h. Scale bar: 400 μm. Data are representative of two repeated experiments. **c**, Cumulative release of verteporfin from fiber for short-term and long-term purposes (*n* = 4). Data are medians ± quartiles. **d**, Viability of 4T1 cancer cells (MTT assay) when treated with verteporfin for 4 h, washed and with or without 20 s light irradiation, and further incubated for 24 or 48 h (*n* = 7). Data are means ± standard deviations.

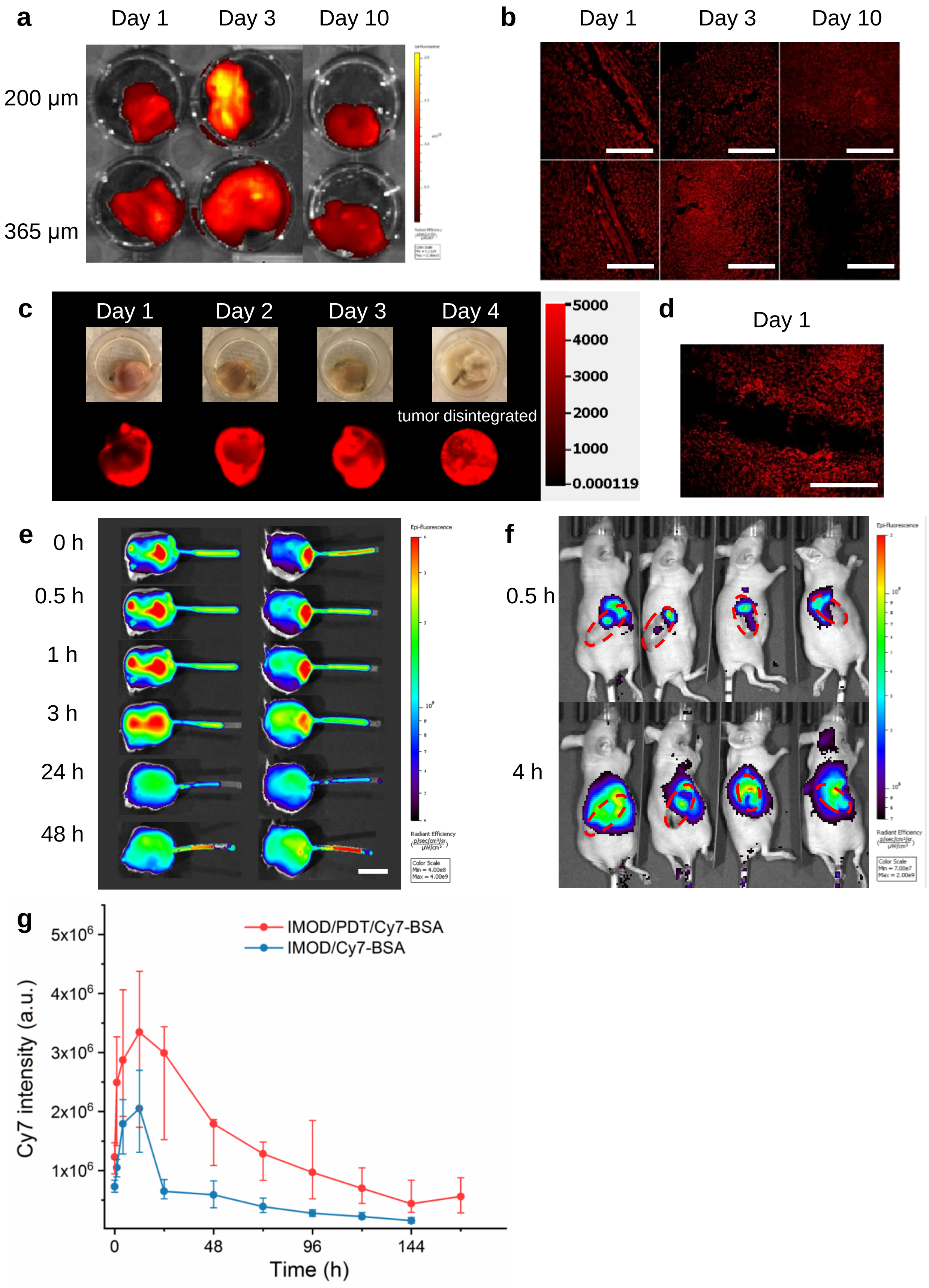
a
Day 1
Day 3
Day 10
200 µm
365 µm
b
Day 1
Day 3
Day 10
c
Day 1
Day 2
Day 3
Day 4
tumor disintegrated
5000
4000
3000
2000
1000
0.000119
d
Day 1
e
0 h
0.5 h
1 h
3 h
24 h
48 h
f
0.5 h
4 h
g
IMOD/PDT/Cy7-BSA
IMOD/Cy7-BSA
Cy7 intensity (a.u.)
5x10^6
4x10^6
3x10^6
2x10^6
1x10^6
0
48
96
144
Time (h)

Supplementary Figure 2. ***Ex vivo*** **intratumoral release of fibers loaded with different drugs.** **a**, Rhodamine loaded fibers (two different diameters) were inserted into excised 4T1 s.c. tumor tissue and allowed to diffuse for 1 to 10 days in Opti-MEM culture medium at 37 °C. **b**, Resulting fluorescent images corresponding to experimental conditions in **a** showing diffused rhodamine within tumor tissue. Scale bar: 400 µm. Data are representative of two repeated experiments. **c**, The verteporfin loaded fibers (365 µm diameter) was inserted into excised 4T1 s.c. tumor tissue and allowed to diffuse for 1 to 4 days in Opti-MEM culture medium at 37 °C. Note that the tumor disintegrated at day 4, presumably due to phototoxicity of verteporfin (light for imaging could activate verteporfin to induce cytotoxicity). **d**, Resulting fluorescent image corresponding to day 1 in **c**, showing verteporfin diffusion in the tumor tissue. Scale bar: 400 µm. Data are representative of two repeated experiments. **e**, Cy5-BSA loaded fiber was inserted into excised 4T1 s.c. tumor tissue and left to diffuse for 48 h in Opti-MEM culture medium at 37 °C. The tip of the fiber outside the tumor was sealed. Scale bar: 2 mm. **f**, IVIS image shows *in vivo* diffusion of verteporfin within tumor tissue at 0.5 h and 4 h post-implantation. Tumor position was circled in red dash lines. **g**, Time course of intratumorl Cy7 intensity (arbitrary unit a.u.) over 1 week (n = 5) for mice studies in Figure 3. Data are medians ± quartiles.

Discussion:

Upon injection, we noticed that the fluorescence signals were different and low in both groups, due to the self-quenching of the Cy7 dye. At 1-12 h post injection, the fluorescence intensity in tumor regions of both groups gradually increased, presumably due to the diffusion of the dye throughout the tumor. The self-quenching phenomena were more pronounced in the group of free Cy7-BSA, likely because diameter of the needle (0.184 mm diameter for 28G needle) for i.t. injection is smaller than that of the IMOD fiber (~ 0.3-0.5 mm), and that the injected solution was more concentrated at the i.t. injection site. Note that verteporfin did not have any substantial absorbance at 800 nm wavelength that can affect the Cy7 fluorescence readings.

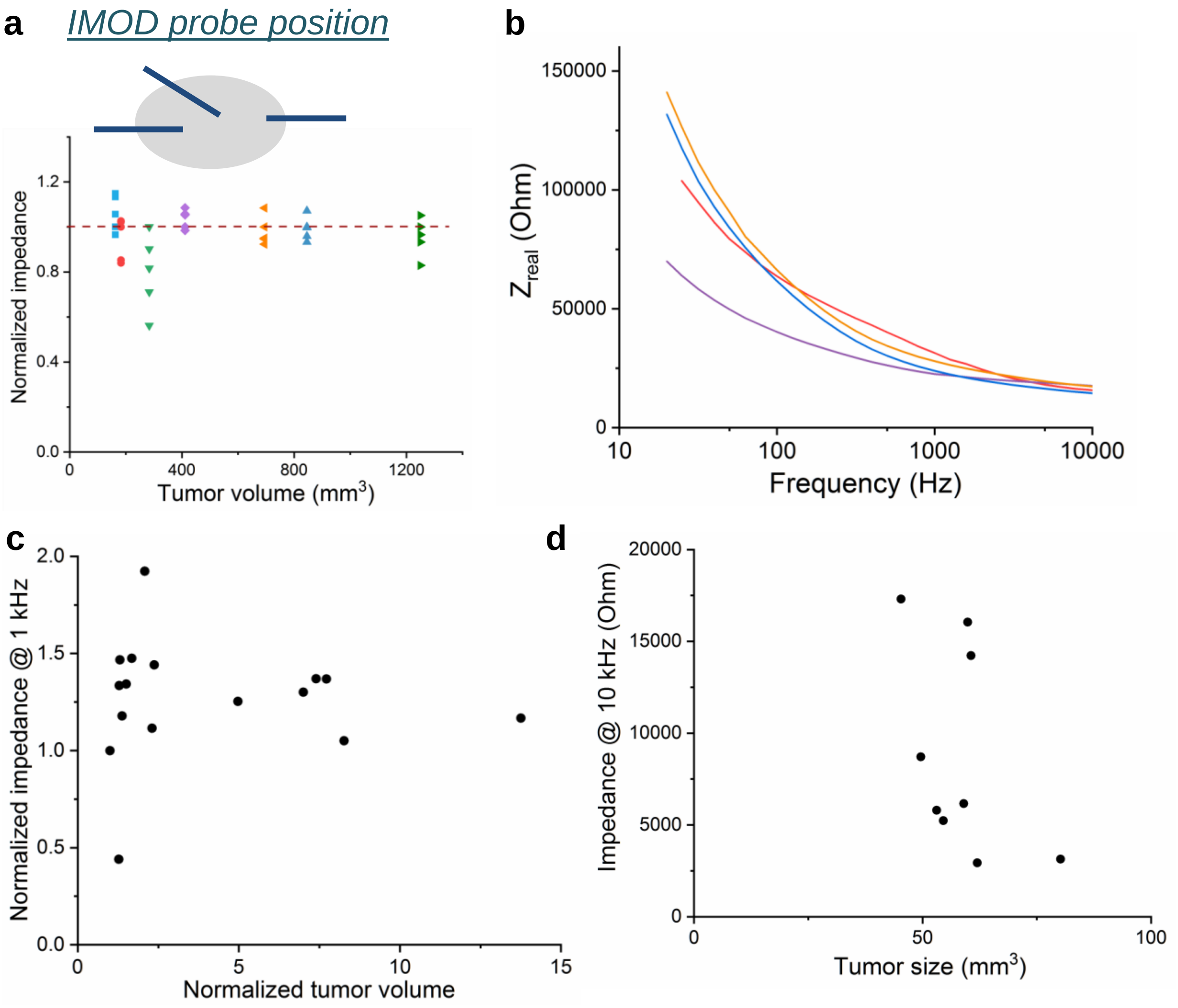

Supplementary Figure 3. The use of IMOD for tumor impedance measurement. a, Relationship between position of fiber-optic probe with respect to tumor core and corresponding impedance measurement. *Ex vivo* tumor impedance measured on excised s.c. 4T1 tumor with various tumor volume. Every data point within the same tumor volume was taken at different positions on the tumor. Each color represents the independent study on each individual tumor volume. **b**, Bode phase impedance plot of s.c. 4T1 tumor over 0.1-10 kHz frequency. The color represents each independent impedance measurement. **c**, No linear relationship between normalized impedance at 1 kHz and normalized tumor volume. **d**, The absolute value of impedance at 10 kHz frequency varied significantly with similar E0771 tumor volume sizes in different mice implanted with IMOD device. Normalized impedance value at 10 kHz was therefore based on the initial impedance measurement value when the device was implanted.

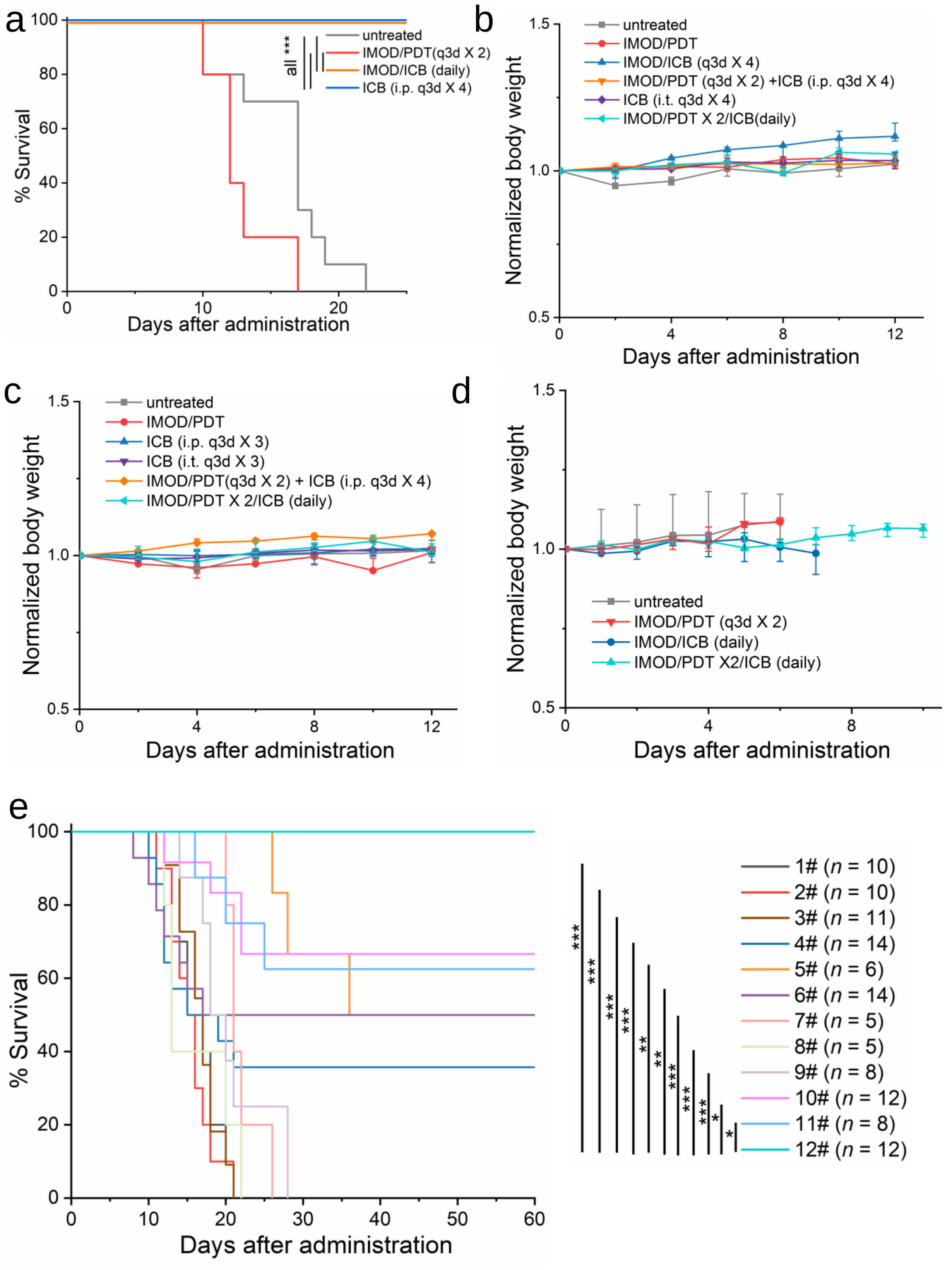

a
% Survival
Days after administration
all ***
untreated
IMOD/PDT(q3d X 2)
IMOD/ICB (daily)
ICB (i.p. q3d X 4)
b
Normalized body weight
Days after administration
untreated
IMOD/PDT
IMOD/ICB (q3d X 4)
IMOD/PDT (q3d X 2) +ICB (i.p. q3d X 4)
ICB (i.t. q3d X 4)
IMOD/PDT X 2/ICB(daily)
c
Normalized body weight
Days after administration
untreated
IMOD/PDT
ICB (i.p. q3d X 3)
ICB (i.t. q3d X 3)
IMOD/PDT(q3d X 2) + ICB (i.p. q3d X 4)
IMOD/PDT X 2/ICB (daily)
d
Normalized body weight
Days after administration
untreated
IMOD/PDT (q3d X 2)
IMOD/ICB (daily)
IMOD/PDT X2/ICB (daily)
e
% Survival
Days after administration
1# (n = 10)
2# (n = 10)
3# (n = 11)
4# (n = 14)
5# (n = 6)
6# (n = 14)
7# (n = 5)
8# (n = 5)
9# (n = 8)
10# (n = 12)
11# (n = 8)
12# (n = 12)

Supplementary Figure 4. IMOD delivers treatments with negligible side effects. a, Survival of BALB/c mice with s.c. CT26 tumors (*n* = 10 for all groups). Weight gain in **b** C57BL/6 mice with s.c. E0771 tumors (*n* = 6-14, see Fig. 4a for *n* of each group); **c** BALB/c mice with s.c. 4T1 tumors (*n* = 7-11, see Fig. 4c for *n* of each groups); and **d** C57BL/6 mice with s.c. B16F10 tumors (*n* = 7-12, see Fig. 4d for *n* of each group). **e**, Treatments performed in subcutaneous (s.c.) E0771 tumors in C57BL/6 mice as supplementary to Figure 4a. Groups: 1#, untreated; 2#, IMOD/PDT (q3d × 2); 3#, ICB (i.p. q3d × 4); 4#, IMOD/ICB (q3d × 4); 5#, IMOD/PDT (q3d × 2) + ICB (i.p. q3d × 4); 6#, ICB (i.t. q3d × 4); 7#, PDT (i.p. q3d × 2) + ICB (i.p. q3d × 4); 8#, PDT (i.p. q3d × 2) + ICB (i.t. q3d × 4); 9#, ICB (i.p. daily); 10#, IMOD/PDT (q3d × 2)/ICB (q2d); 11#, IMOD/PDT (q3d × 2) + ICB (i.p. daily); 12#, IMOD/PDT (q3d × 2)/ICB(daily). The *n* numbers for all groups were labeled in the figure. Data in **b**, **c**, **d** are medians ± quartiles. In **a** and **e**, statistical analysis is performed using log-rank test. * $P < 0.05$, ** $P < 0.01$, *** $P < 0.001$. Source data and *P* values in **a** and **e** are provided in the Source data file.

Discussion

The immunosuppressive tumor microenvironment requires constant reinvigoration of T cells to avoid terminally exhaustion. For IMOD/PDT with i.p. injected ICB antibodies, the tumor concentration of ICB antibodies are expected to be significantly lower than those via locally administered ICB with high frequency. The i.p treatments of ICB antibodies may not be sufficient to reverse the T cell exhaustion course in tumor: our flow cytometry studies showed that i.p. treatment with ICB antibodies increased the number of $CD8^+$ TILs (Supplementary Fig. 10a), but resulted in low levels of proliferative markers (compared with the levels in other groups, Fig. 7a), and high fractions of terminally exhausted subsets of $CD8^+$ TILs (also see recent studies comparing i.p and i.t distribution of ICB antibodies[2]), which was all reflected in the groups receiving daily i.p. or q3d × 4 i.p ICB antibodies in **e**.

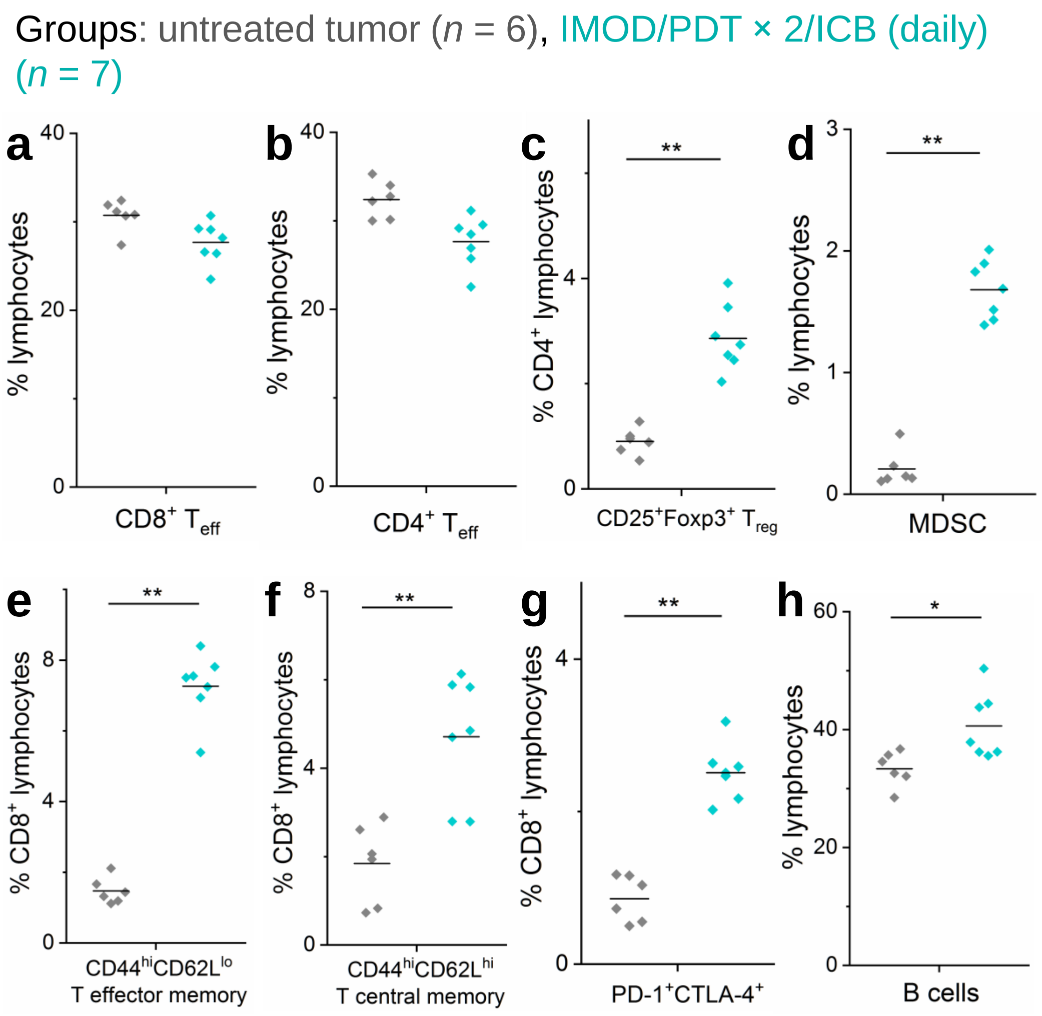

Supplementary Figure 5. The combination treatment of PDT and ICB antibodies via IMOD elicits lymphocytes changes in lymph nodes of C57BL/6 mice after rechallenging by subcutaneously injected E0771 tumor cells. Initial E0771 tumors in C57BL/6 mice were cured and rechallenged on day 80 (the study in Figure 4a). All mice rejected the E0771 cells and their lymph nodes were collected on day 90 after the start of treatment and analyzed by flow cytometry, compared to mice without any treatment (*n* = 6 for -7). Percentages of **a** $CD8^+CD3^+$ effector T cells (T_{eff}s) in lymphocytes; **b** $CD4^+CD3^+$ T_{eff}s in lymphocytes; **c** $CD25^+Foxp3^+$ regulatory T cells (T_{reg}s) in $CD4^+$ lymphocytes; **d** $CD11b^+Ly6G^-Ly6C^{hi}$ myeloid-derived suppressor cells (MDSCs) in lymphocytes; **e** $CD44^{hi}CD62L^{lo}$ T effector memory cells in $CD8^+$ lymphocytes; **f** $CD44^{hi}CD62L^{hi}$ T central memory cells in $CD8^+$ lymphocytes; **g** $PD\text{-}1^+CTLA\text{-}4^+$ cells in $CD8^+$ lymphocytes; **h** $CD19^+$ B cells in lymphocytes. Statistical analyses are performed using Mann-Whitney *U*-test. * $P < 0.05$, ** $P < 0.01$. Source data and *P* values are provided in the Source data file.

Discussion:

Though cured mice did not exhibit significant change in the fraction of $CD8^+$ and $CD4^+$ effector T cells in lymph nodes upon rechallenge (Supplementary Fig. 5a, b), a significant increase in both effector and central memory $CD8^+$ T cells in the lymph nodes on day 90 was observed (P = 0.001 and 0.004, respectively; n = 6 or 7, Supplementary Fig. 5e, f). The increased fractions of T_{reg}s and MDSCs were likely due to immunosuppressive effects induced by injected tumor cells (Supplementary Fig. 5c, d). The increased fraction of double positivie $CTLA^+PD\text{-}1^+$ in $CD8^+$ T cells suggested that $CD8^+$ T cells were activated to combat tumor cells (Supplementary Fig. 5g). The increased fractions of B cells may indicate its role in assisting $CD8^+$ and $CD4^+$ T cells against tumor.[3, 4]

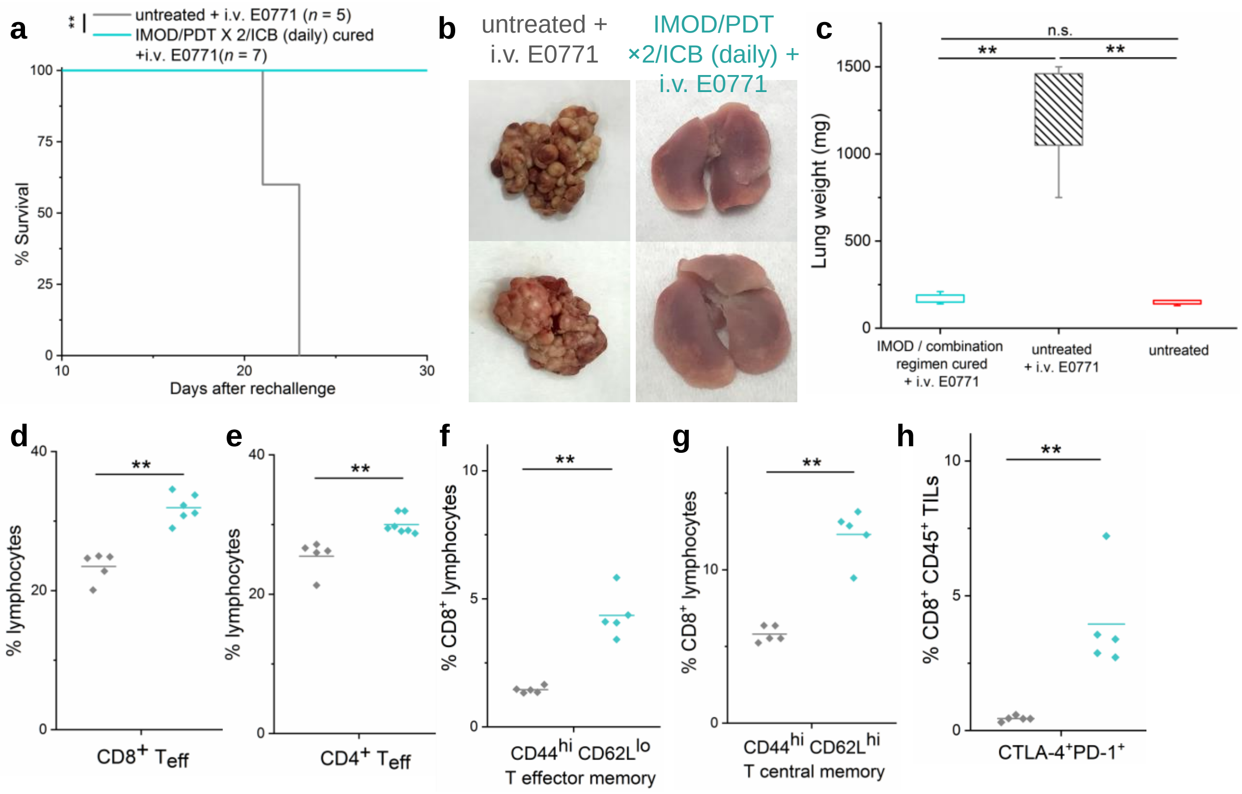

Supplementary Figure 6. The combination treatment of PDT and ICB antibodies via IMOD elicits anti-tumor immunological memory in lymphocytes in C57BL/6 mice upon rechallenging by intravenously (i.v.) injected E0771 tumor cells. Initial E0771 tumors in C57BL/6 mice were cured by the combination regimen and were rechallenged on day 60 via tail-vein intravenous injection of 10^6 tumor cells. **a**, All E0771-tumor-bearing mice cured by the combination of PDT and ICB antibodies via IMOD could survive and reject tumor growth 30 days after administration (n = 5–7). **b**, Representative lung images comparing untreated mice to cured mice in **a**. All mice were i.v. injected with 10^6 E0771 cells. **c**, Comparison of lung weights from the two groups at the end of the study in **a**, and normal C57BL/6 mice without any treatment. The box indicates the interquartile range; and the upper and lower whiskers show the 95% and 5% percentile values, respectively. Flow cytometry studies were performed with lymphocytes from lymph nodes of mice in **a**. Percentages of **d,** $CD8^+CD3^+$ effector T cells (T_{eff}s) in lymphocytes (n = 5 for untreated, n = 6 for pre-treated with IMOD/PDT×2/ICB(daily)); **e,** $CD4^+CD3^+$ T_{eff}s in lymphocytes (n = 5 for untreated, n = 7 for pre-treated with IMOD/PDT×2/ICB(daily)); **f,** $CD44^{hi}CD62L^{lo}$ T effector memory cells in $CD8^+$ lymphocytes (n = 5 for all groups); **g,** $CD44^{hi}CD62L^{hi}$ T central memory cells in $CD8^+$ lymphocytes (n = 5 for all groups); **h,** PD-1^+CTLA-4^+ cells in $CD8^+$ lymphocytes (n = 5 for all groups). Statistical analyses are performed using Mann-Whitney U-test. * $P < 0.05$, ** $P < 0.01$. Source data and P values in **a** and **c-h** are provided in the Source data file.

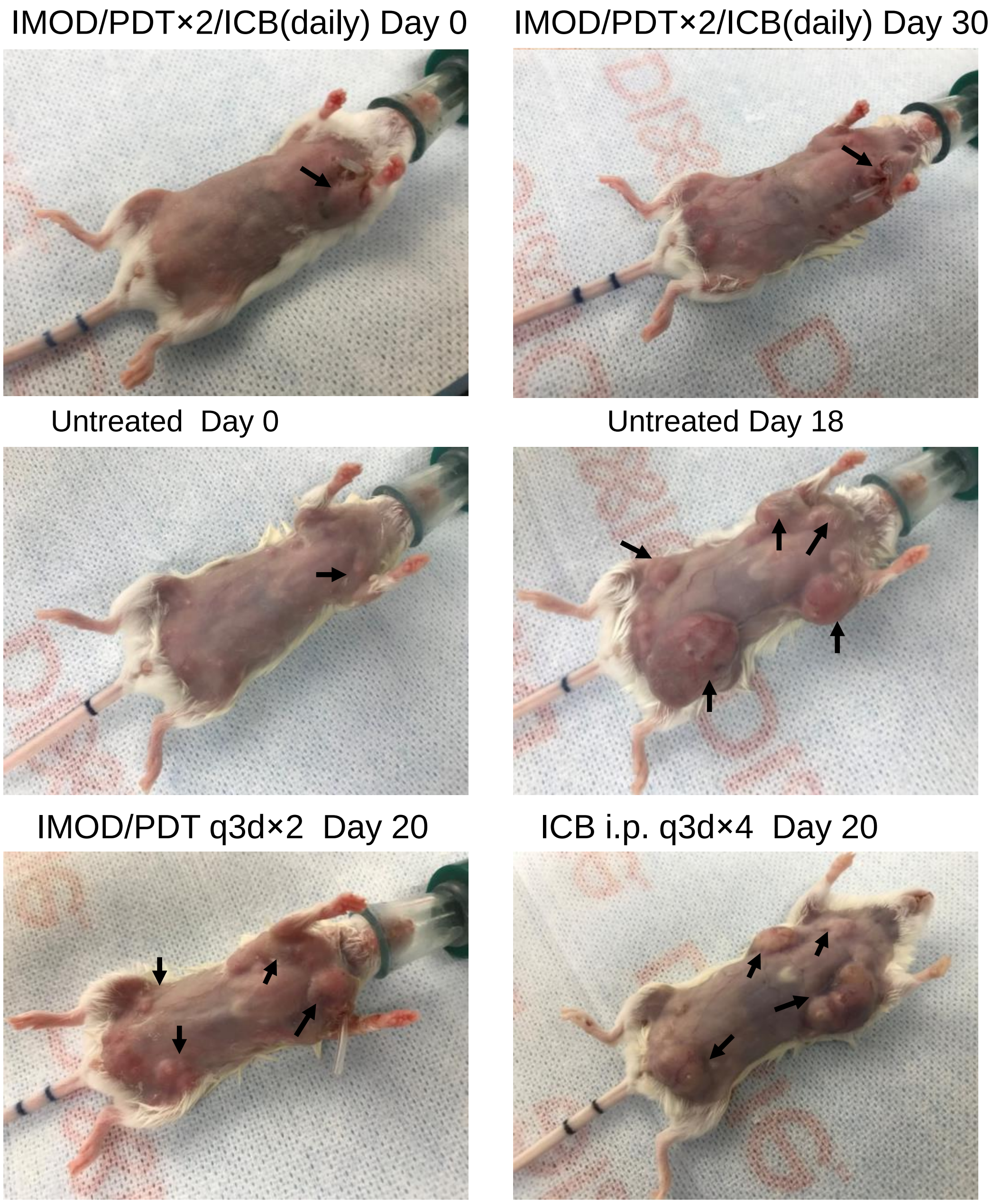

Supplementary Figure 7. The combination treatment of PDT and ICB antibodies via IMOD was therapeutic in a spontaneous tumor model. MMTV-PyMT transgenic female mice were untreated or were treated with PDT (q3d × 2) via implanted IMOD, treated with ICB antibodies (i.p. q3d × 4), or treated with PDT (q3d × 2) and ICB antibodies (daily) via IMOD (referred as IMOD/PDT × 2/ICB (daily)), starting from week 8 of mouse age when mammary tumor sizes were ~ 50 mm^3. Images were taken at different stage of treatment (for survival data, see Figure 4e), and arrows indicate arising mammary tumors (time points differ due to significant tumor burdens and affected mice had to be euthanized following animal protocol). MMTV-PyMT receiving the

combination regimen exhibited minimal contralateral tumor growth on day 30 compared to other groups, even at earlier time points.

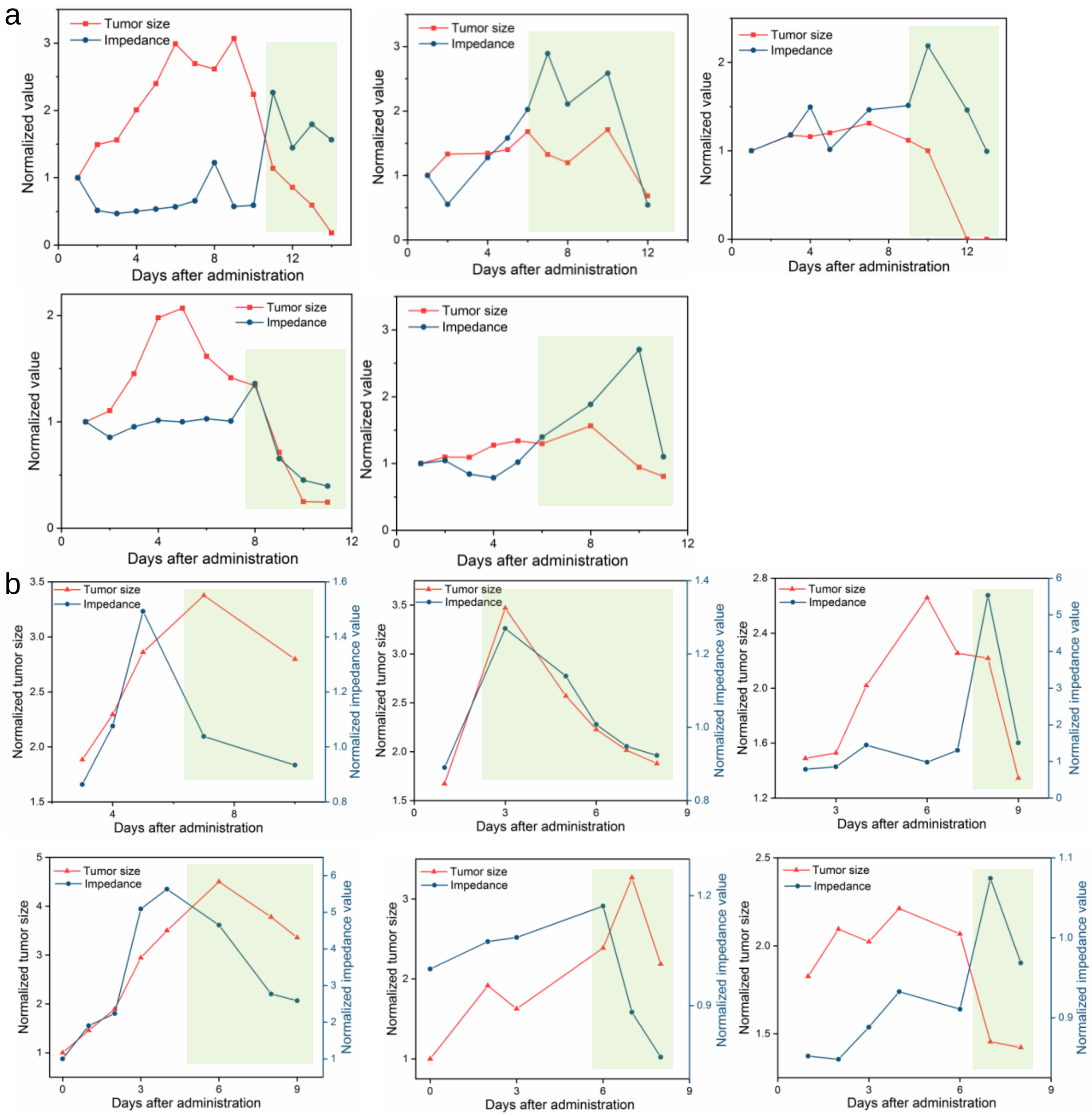

Supplementary Figure 8. Tumor size shrinkage can be correlated with decrease of the measured tumor impedance value (highlighted in light green boxes). a, Results of treating s.c. CT26 tumor through i.p. injections of ICB antibodies, with tumor impedance measured via implanted IMOD. **b**, Results of treating s.c. E0771 tumor through injections of ICB antibodies via IMOD every two days. Tumor impedance values were measured using IMOD before treatment administration. Source data are provided in the Source data file.

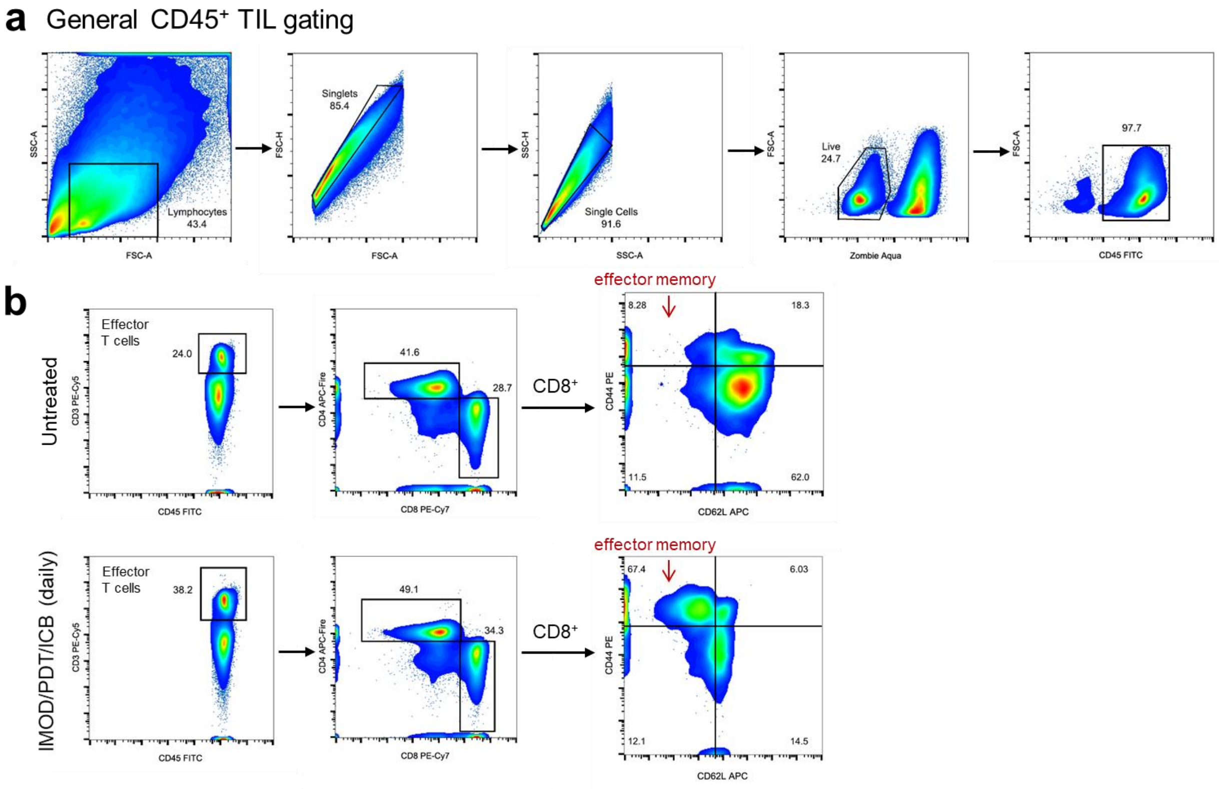

Supplementary Figure 9. Representative flow cytometry gating of TILs in E0771 tumors. a live CD45$^+$ TILs; **b** CD44^{hi}CD62L^{lo} T effector memory cells in CD8$^+$CD3$^+$ TILs. Upregulation of T memory cells observed in E0771 tumors treated with PDT and ICB antibodies delivered daily via IMOD compared to untreated E0771 tumors.

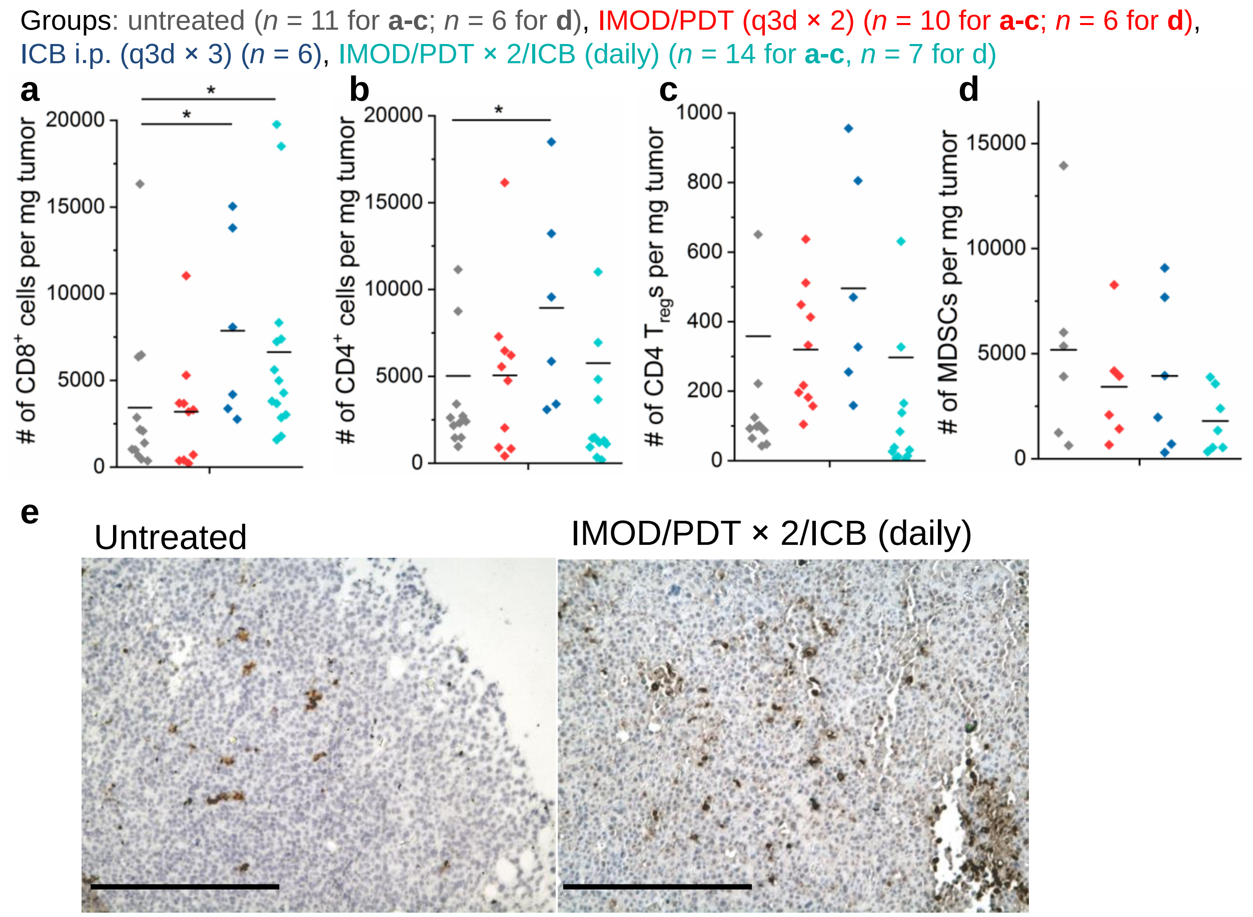

Supplementary Figure 10. Effects of delivering a combination of PDT and ICB antibodies via IMOD on TILs' numbers in E0771 tumors (supplementary to Figure 6). E0771 tumors (*n* = 6-14) in C57BL/6 mice were untreated, or treated with PDT (q3d × 2) via IMOD, ICB antibodies (i.p. q3d × 3), or PDT (q3d × 2) and ICB antibodies (daily) via IMOD (referred to as IMOD/PDT × 2/ICB (daily)). Tumors were collected on day 9-10 after the start of treatment and analyzed by flow cytometry. **a**, Numbers of $CD8^+CD3^+$ effector T cells (T_{eff}s) in TILs. **b**, Numbers of $CD4^+CD3^+$ T_{eff}s in TILs. **c**, Numbers of $CD4^+CD25^+Foxp3^+$ regulatory T cells (T_{reg}s) in TILs. **d**, Numbers of $CD11b^+Ly6G^-Ly6C^{hi}$ myeloid-derived suppressor cells (MDSCs) in TILs. Statistical analyses are performed using Mann-Whitney *U*-test. * $P < 0.05$. **e**, Representative IHC images of $CD8^+$ expression on TILs observed in E0771 tumors that were either untreated or treated with PDT plus ICB antibodies via IMOD (brown cells indicate positively-stained cells). Scale bar: 400 µm. Images are representative of two repeated experiments. Source data and *P* values in **a-d** are provided in the Source data file.

Discussion:

Both PD-1 and CTLA-4 immune checkpoint molecules are expressed/upregulated a few hours after $CD8^+$ T cells are activated, and start to promote $CD8^+$ T cells exhaustion. ICB antibodies bind to corresponding checkpoint molecules, preventing the checkpoint molecules from binding to CD28 and other ligands (PD-L1 etc.), which would attenuate positive T cell co-stimulation signals.[5, 6] The ICB antibodies cannot decrease or down-regulate the expression of CTLA-4 and PD-1. Instead, it is reported that the administration of ICB antibodies (or vaccines) does increase the frequency of double positive $CD8^+$ T cells (also seen in our Fig. 6i), which indicates that more naïve $CD8^+$ T cells are activated after treating with ICB antibodies.[7] This also suggests the importance of continuously administrating ICB antibodies in order to prevent $CD8^+$ T cells from exhaustion.[7, 8]

Groups: mice without tumor (*n* = 6), untreated tumor (*n* = 12 in **a**, **b**, **f**, **g**; *n* = 6 in the rest), IMOD/PDT (q3d × 2) (*n* = 9 in **a**, **b, f**, **g**; *n* = 5 in the rest) , ICB i.p. (q3d × 3) (*n* = 6), IMOD/PDT × 2/ICB (daily) (*n* = 11 in **a**, **b**, **f**, **g**; *n* = 7 in the rest)

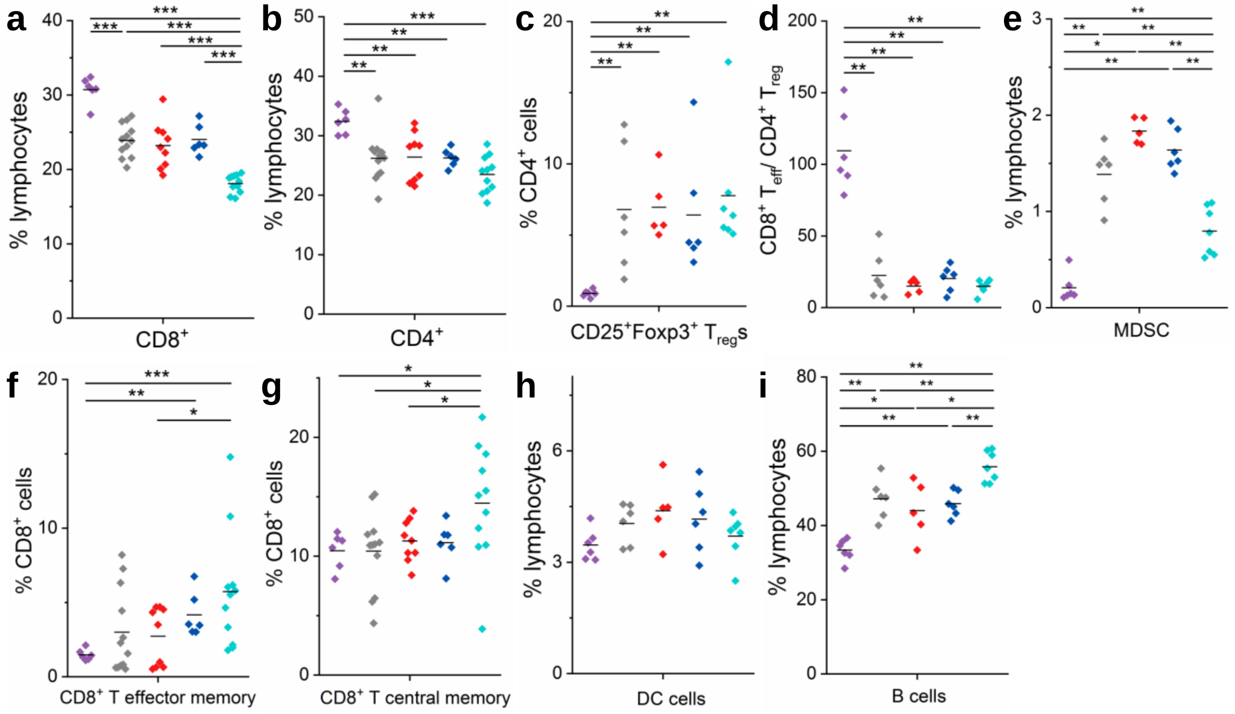

Supplementary Figure 11. Effects of delivering a combination of PDT and ICB antibodies via IMOD on lymphocytes in tumor-draining lymph nodes of E0771 tumor-bearing C57BL/6 mice. E0771 tumor-bearing C57Bl/6 mice were untreated, or treated with PDT (q3d × 2) via IMOD, ICB antibodies (i.p. q3d × 3), or PDT (q3d × 2) and ICB antibodies (daily) via IMOD (referred to as IMOD/PDT × 2/ICB (daily)). Tumor-draining lymph nodes (*n* = 5-12) were collected on day 9-10 after the start of treatment and analyzed by flow cytometry. **a**, Frequencies of $CD8^+CD3^+$ T_{eff}s in lymphocytes. **b**, Frequencies of $CD4^+CD3^+$ T_{eff}s in lymphocytes. **c**, Frequencies of $CD25^+Foxp3^+$ T_{reg}s in $CD4^+$ lymphocytes. **d**, Ratio of $CD8^+CD3^+$ T_{eff}s to $CD4^+CD25^+Foxp3^+$ T_{reg}s. **e**, Frequencies of $CD11b^+Ly6G^-Ly6C^{hi}$ myeloid-derived suppressor cells (MDSCs) in lymphocytes. **f**, Frequencies of $CD44^{hi}CD62L^{lo}$ effector memory cells in $CD8^+$ lymphocytes. **g**, Frequencies of $CD44^{hi}CD62L^{hi}$ central memory cells in $CD8^+$ lymphocytes. **h**, Frequencies of $CD11c^+$ dendritic cells in lymphocytes. **i**, Frequencies of $CD19^+$ B cells in lymphocytes. Statistical analyses are performed using Mann-Whitney *U*-test. * $P < 0.05$, ** $P < 0.01$, *** $P < 0.001$. Source data and *P* values are provided in the Source data file.

Groups: untreated (*n* = 7), IMOD/PDT (q3d × 2) (*n* = 6), ICB i.p. (q3d × 3) (*n* = 11), IMOD/PDT × 2/ICB (daily) (*n* = 12)

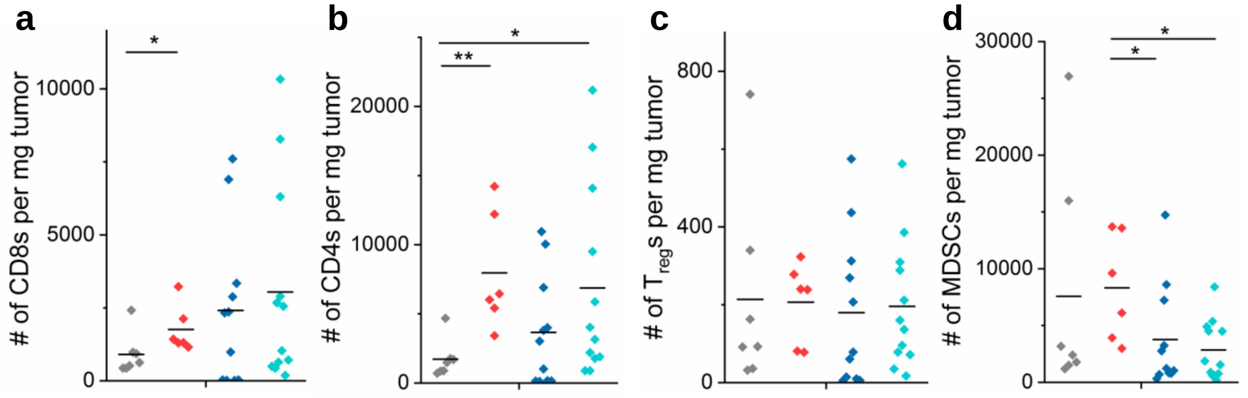

Supplementary Figure 12. Effects of delivering a combination of PDT and ICB antibodies delivery via IMOD on numbers of TILs in 4T1 tumors. 4T1 tumors (*n* = 6-12) in BALB/c mice were untreated, or treated with PDT (q3d × 2) via IMOD, ICB antibodies (i.p. q3d × 3), or PDT (q3d × 2) and ICB antibodies (daily) via IMOD (referred to as IMOD/PDT × 2/ICB (daily)). Tumors were collected on day 9-10 after the start of treatment and analyzed by flow cytometry. **a**, Numbers of CD8$^+$CD3$^+$ T$_{eff}$s in TILs. **b**, Numbers of CD4$^+$CD3$^+$ T$_{eff}$s in TILs. **c**, Numbers of CD4$^+$CD25$^+$Foxp3$^+$ T$_{reg}$s in TILs. **d**, Numbers of CD11b$^+$Ly6G$^-$Ly6C^{hi} MDSCs in TILs. Statistical analyses are performed using Mann-Whitney *U*-test. * $P < 0.05$, ** $P < 0.01$. Source data and *P* values are provided in the Source data file.

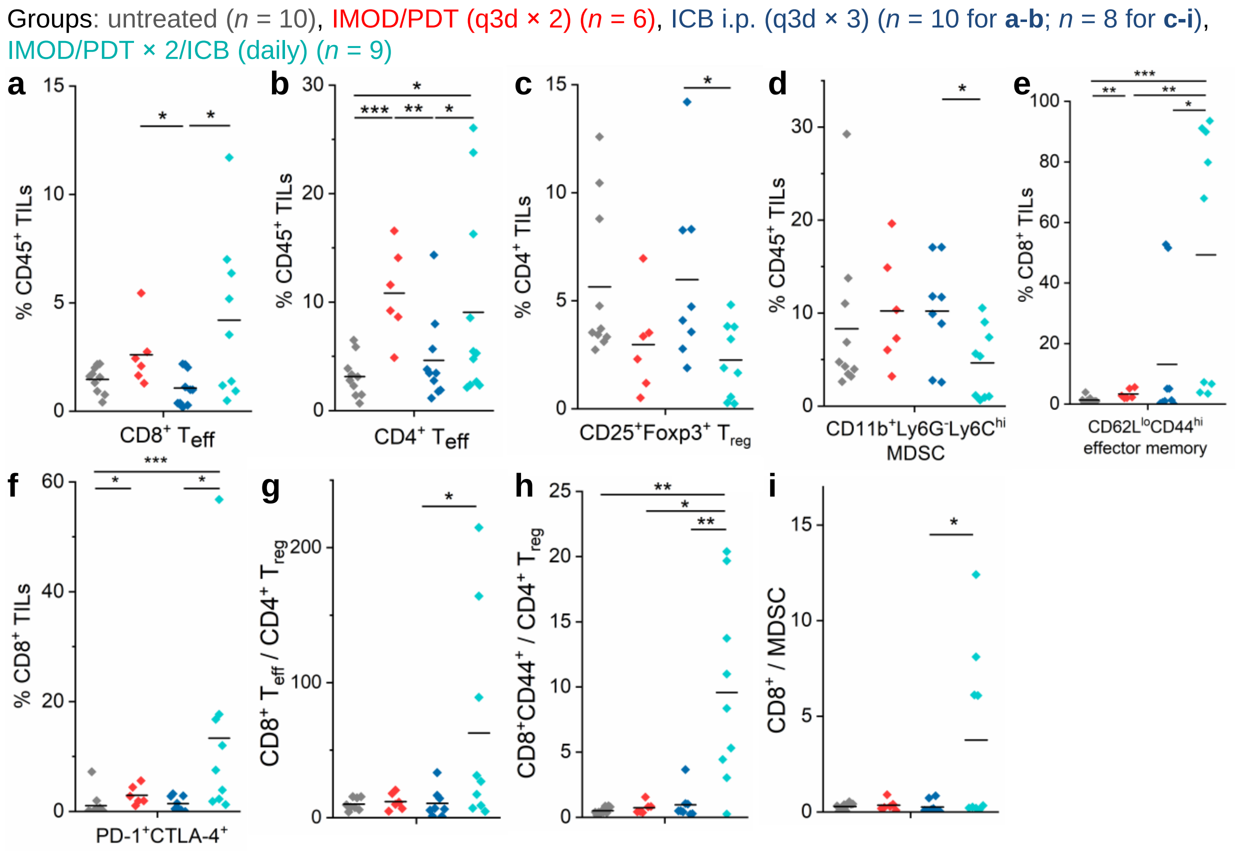

Supplementary Figure 13. Effects of delivering a combination of PDT and ICB antibodies via IMOD on TILs in 4T1 tumor. 4T1 tumors (*n* = 6-10) in BALB/c mice were untreated, or treated with PDT (q3d × 2) via IMOD, ICB antibodies (i.p. q3d × 3), or PDT (q3d × 2) and ICB antibodies (daily) via IMOD (referred to as IMOD/PDT × 2/ICB (daily)). Tumors were collected on day 9-10 after the start of treatment and analyzed by flow cytometry. **a**, Frequencies of $CD8^+CD3^+$ T_{eff}s in TILs. **b**, Frequencies of $CD4^+CD3^+$ T_{eff}s in TILs. **c**, Frequencies of $CD25^+Foxp3^+$ T_{reg}s in $CD4^+$ TILs. **d**, Frequencies of $CD11b^+Ly6G^-Ly6C^{hi}$ MDSCs in TILs. **e**, Frequencies of $CD44^{hi}CD62L^{lo}$ effector memory cells in $CD8^+$ TILs. **f**, Frequencies of $PD\text{-}1^+CTLA\text{-}4^+$ cells in $CD8^+$ TILs. **g**, The ratio of $CD8^+$ T_{eff}s to $CD4^+CD25^+Foxp3^+$ T_{reg}s. **h**, The ratio of $CD8^+CD44^+$ T cells to $CD4^+CD25^+Foxp3^+$ T_{reg}s. **i**, The ratio of $CD8^+CD3^+$ T_{eff}s to MDSC. Statistical analyses are performed using Mann-Whitney *U*-test. * $P < 0.05$, ** $P < 0.01$, *** $P < 0.001$. Source data and *P* values are provided in the Source data file.

Groups: mice without tumor (n = 6), untreated tumor (n =8), IMOD/PDT (q3d × 2) (n = 6), ICB i.p. (q3d × 3) (n = 7), IMOD/PDT × 2/ICB (daily) (n = 7)

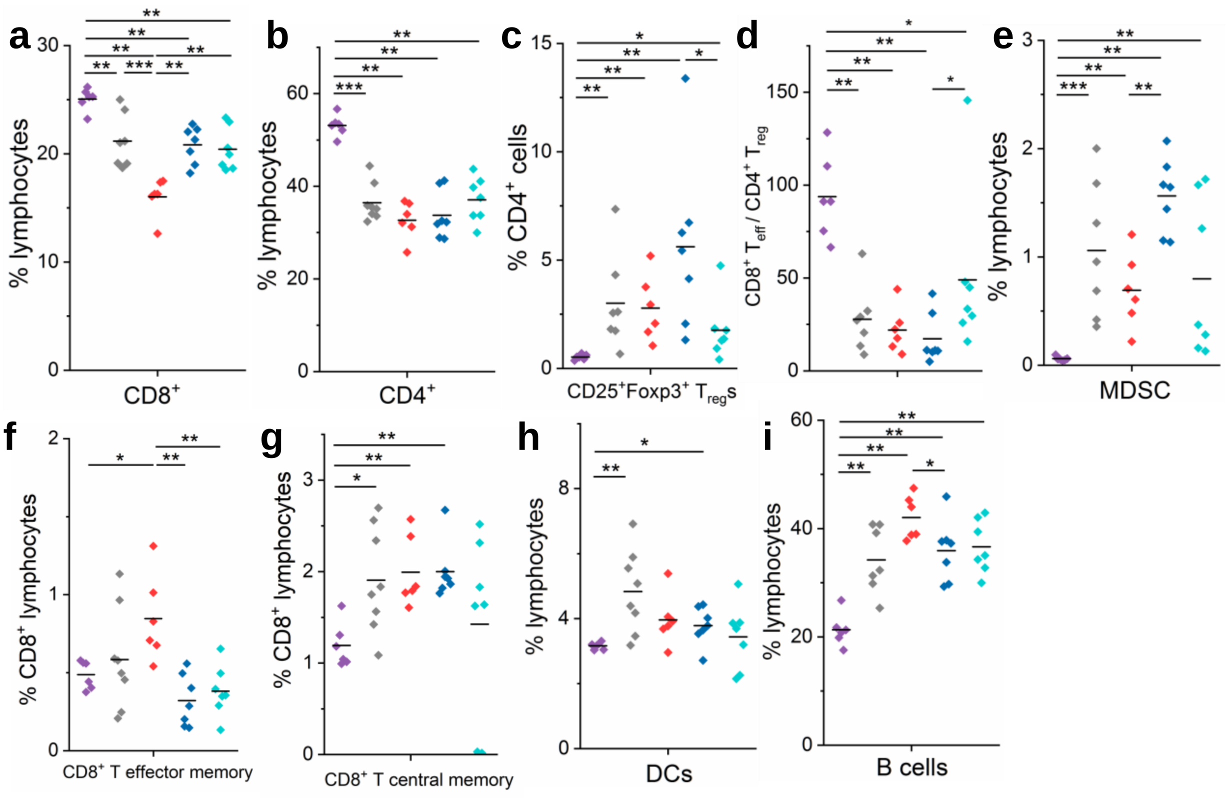

Supplementary Figure 14. Effects of delivering a combination of PDT and ICB antibodies via IMOD on lymphocytes in tumor-draining lymph nodes of 4T1 tumor-bearing BALB/c mice. 4T1 tumor-bearing BALB/c mice were untreated, or treated with PDT (q3d × 2) via IMOD, ICB antibodies (i.p. q3d × 3), or PDT (q3d × 2) and ICB antibodies (daily) via IMOD (referred to as IMOD/PDT × 2/ICB (daily)). Tumor-draining lymph nodes (n = 6-8) were collected on day 9-10 after the start of treatment and analyzed by flow cytometry. **a**, Frequencies of $CD8^+CD3^+$ T_{eff}s in lymphocytes. **b**, Frequencies of $CD4^+CD3^+$ T_{eff}s in lymphocytes. **c**, Frequencies of $CD25^+Foxp3^+$ T_{reg}s in $CD4^+$ lymphocytes. **d**, Ratio of $CD8^+CD3^+$ T_{eff}s to $CD4^+CD25^+Foxp3^+$ T_{reg}s. **e**, Frequencies of $CD11b^+Ly6G^-Ly6C^{hi}$ MDSCs in lymphocytes. **f**, Frequencies of $CD44^{hi}CD62L^{lo}$ effector memory cells in $CD8^+$ lymphocytes. **g**, Frequencies of $CD44^{hi}CD62L^{hi}$ central memory cells in $CD8^+$ lymphocytes. **h**, Frequencies of $CD11c^+$ dendritic cells in lymphocytes. **i**, Frequencies of $CD19^+$ B cells in lymphocytes. Statistical analyses are performed using Mann-Whitney U-test. * $P < 0.05$, ** $P < 0.01$, *** $P < 0.001$. Source data and P values are provided in the Source data file.

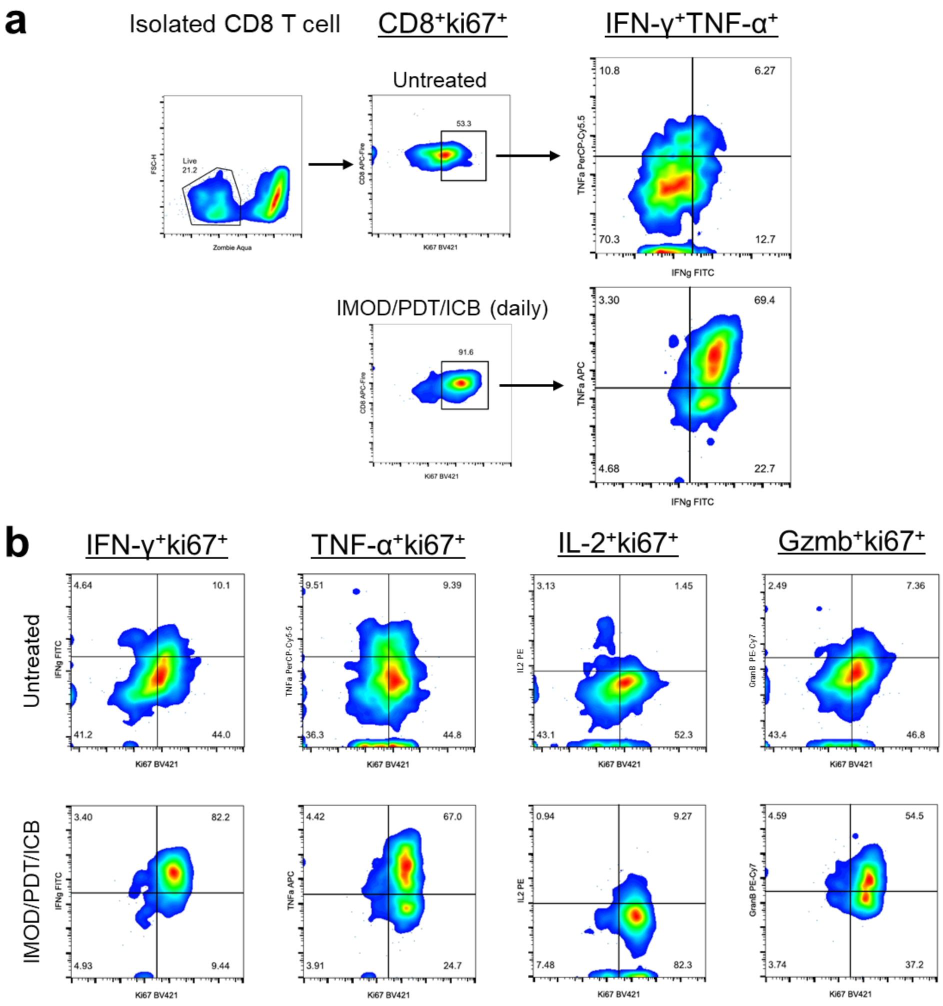

Supplementary Figure 15. Representative flow cytometry gating of isolated CD8$^+$ TILs. **a**, live and stimulated CD8$^+$ TILs, and the comparison of proliferative marker ki-67 levels, and polyfunctional subsets having IFN-γ$^+$TNF-α$^+$ cytokines between treated (PDT plus ICB antibodies) and untreated tumors. **b,** Representative results showing the increase in proliferation and cytotoxic activity of proliferative CD8$^+$ TILs secreting IFN-γ, TNF-α, IL-2, and granzyme B cytokines.

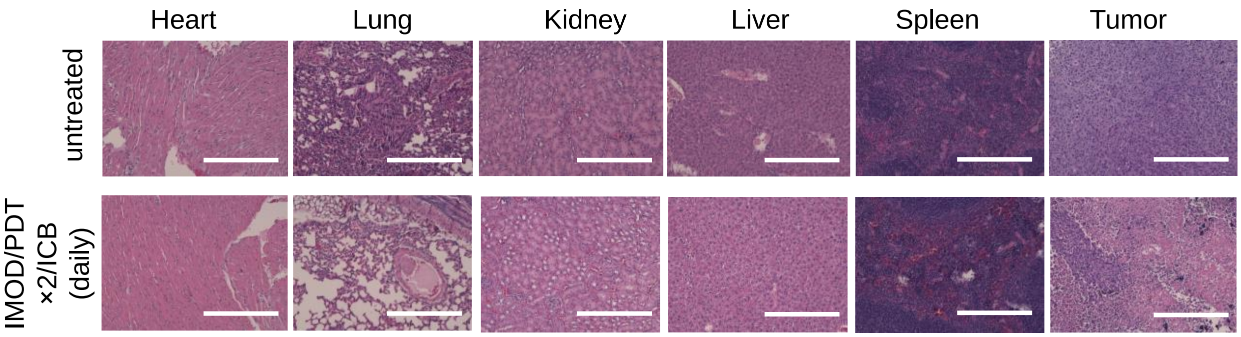

Supplementary Figure 16. Representative haematoxylin and eosin stained sections of major organs and tumor collected on day 7 after start of treatment from E0771 tumor-bearing C57BL/6 mice that were untreated and administered with PDT/ICB/IMOD. Combined treatment of PDT and daily dosing of ICB antibodies via IMOD in E0771 tumors in C57BL/6 mice did not present toxicities on major organs. Note that mice receiving combination regimen were cured around 10-14 days after the start of the treatment; for prolonged tissue histology studies, see Supplementary Figure 17. Scale bar: 400 µm. Data are representative of two repeated experiments.

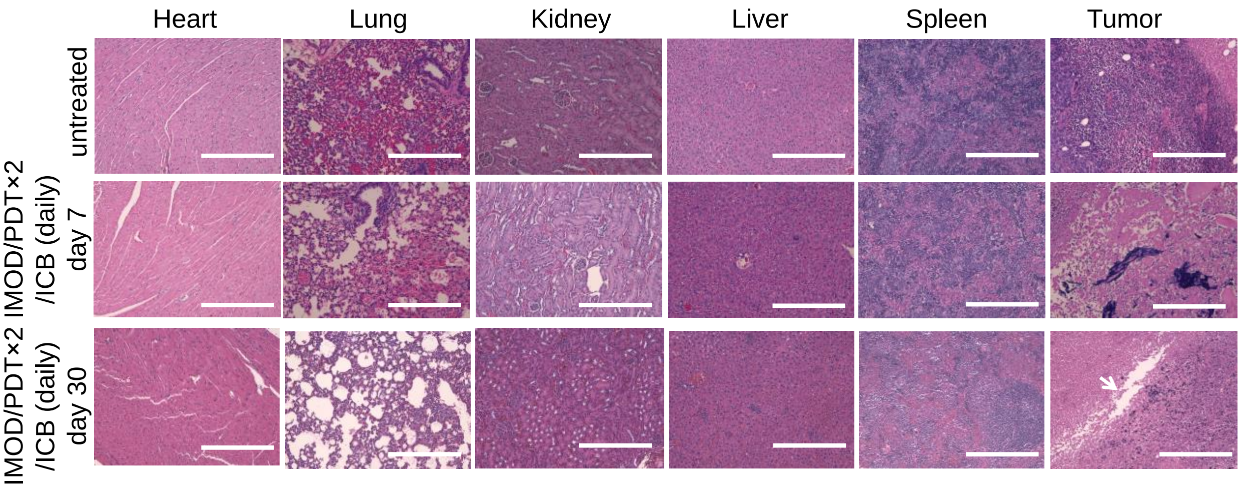

Supplementary Figure 17. Representative haematoxylin and eosin stained sections of major organs and tumor collected on day 7 and day 30 after start of treatment from 4T1 tumor-bearing BALB/c mice that were untreated and administered with PDT/ICB/IMOD. Combined treatment of PDT and daily dosing of ICB antibodies via IMOD in 4T1 tumors in BALB/c mice did not exhibit toxicities in major organs. On day 30, no multinucleated foreign body giant cell was found around the site where IMOD fiber was inserted into the tumor (pointed by white arrow), presumably due to the immunosuppressive environment inside the tumor. Scale bar: 400 μm. Data are representative of two repeated experiments.

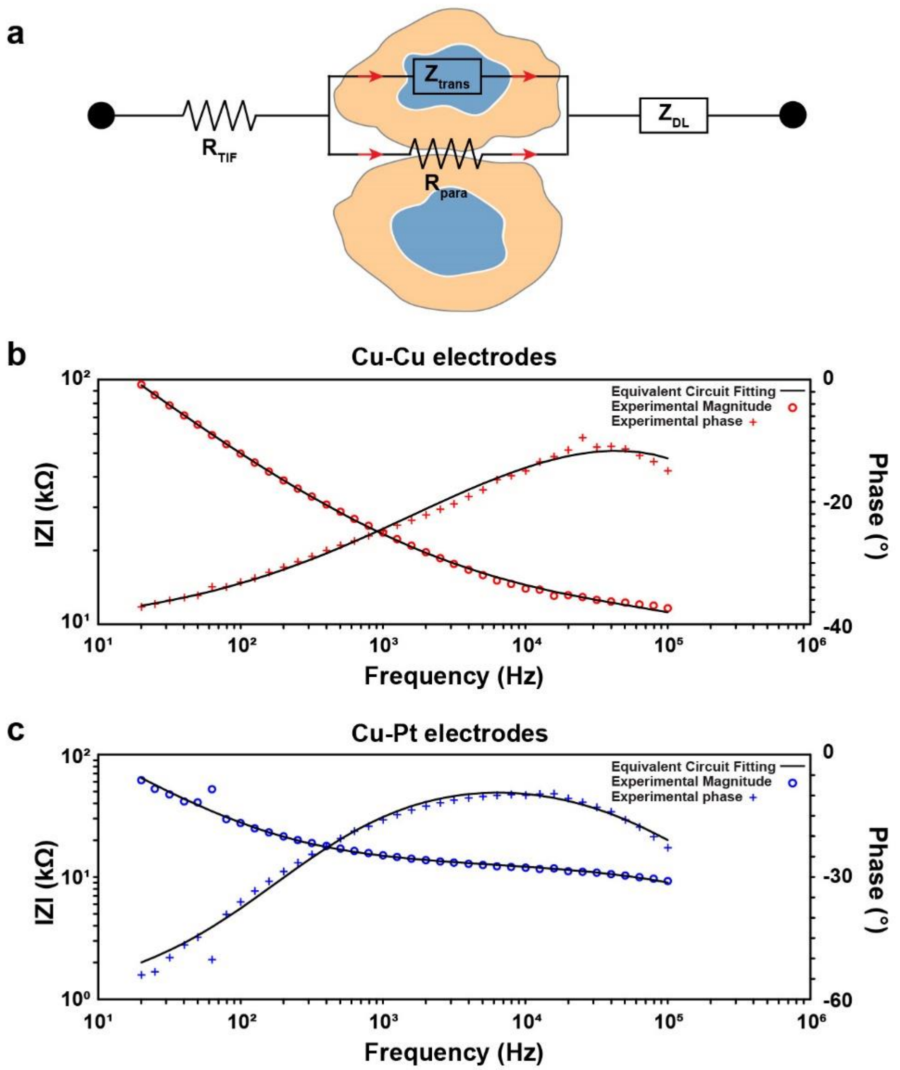

Supplementary Figure 18. Modeling of tumor impedance measurement in E0771 tumors and comparison between experiments and simulation curves. a, Proposed equivalent circuit model for electrode–tumor tissue interface with equation (Circuit Model Discussion, equation 3) that represents the total impedance of the equivalent circuit model. **b**, representative impedance spectra for experimental data of copper/copper electrodes in E0771 tumors (red) and equivalent circuit fitting (black) used for curve fitting. **c**, representative impedance spectra for experimental data of copper/platinum electrodes in E0771 tumors (blue) and equivalent circuit fitting (black) used for curve fitting.

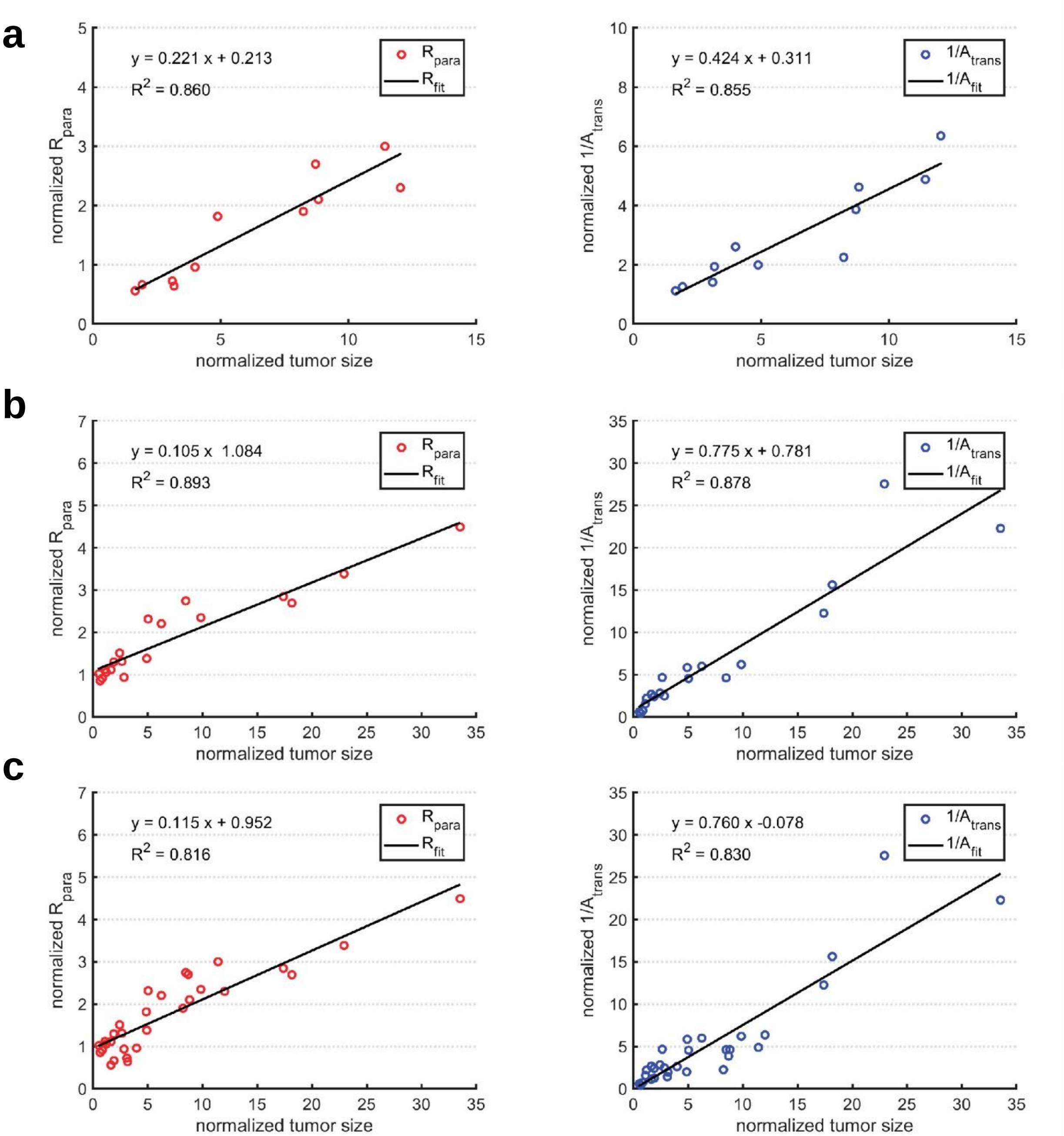

Supplementary Figure 19. Fitting results of E0771 tumor measurements using proposed circuit models (see circuit model discussion section). a-b, Two independent impendance measurements in mice bearing E0771 tumors were used to obtain paracellular resistance (R_{para}) or transcellular impedance ($1/A_{trans}$) values for the circuit model. **c**, Fitting results in **a-b** were combined to show normalized R_{para} and $1/A_{trans}$ increased linearly with the normalized tumor size. Fitting data were calculated and plotted using Echem Analyst™ software by Gamry Instruments.

3. Supplementary Table

Supplementary Table 1. Parameters fitting of equations in different impedance measurement experiments. Equations are shown in the discussion section of circuit model.

	Cu-Cu electrodes		Cu-Pt electrodes	
	mean	SD	mean	SD
R_{TIF} (Ω)	558.33	71.28	554.33	59.57
A_{DL} (S*$S^{n_{DL}}$)	1.27×10^{-6}	0.04×10^{-6}	7.75×10^{-7}	0.76×10^{-7}
n_{DL}	0.45	0.05	0.66	0.01
R_{para} (kΩ)	9.68	0.18	11.67	0.33
A_{trans} (S*$S^{n_{trans}}$)	5.18×10^{-10}	0.44×10^{-10}	1.05×10^{-8}	0.09×10^{-8}
n_{trans}	0.77	0.01	0.64	0.01

4. Circuit Model Discussion

As shown by many researchers, the current predominantly flows through the paracellular space between cells at low frequency and the majority of the current takes the transcellular pathway across the cells when the frequency is over 10 kHz.[9-12] To simulate this current flow, a simplified equivalent circuit model is proposed to gain insights into the obtained impedance spectrum (Supplementary Fig. 18). This circuit model consists of a resistance of the tumor interstitial fluid (TIF, R_{TIF}) in series with a constant phase element (CPE) representing double layer capacitor (Z_{DL}) and a parallel combination of the paracellular resistance (R_{para}) and another CPE simulating the transcellular behavior of the cell (Z_{trans}).

The impedance of the constant phase element is $\frac{1}{A(j\omega)^n}$, where $i = \sqrt{-1}$, $0 < n < 1$. The angular frequency $\omega = 2\pi f$ with f as frequency in Hz, and A is the CPE admittance at $\omega = 1$ rad/s. The parameter n is such that if n = 1, the impedance of a CPE is that of an ideal capacitor; and when n = 0, the CPE is a pure resistor. Applying this principle to the constant phase elements in the circuit results in their impedance as $Z_{DL} = \frac{1}{A_{DL}(j\omega)^{n_{DL}}}$ and $Z_{trans} = \frac{1}{A_{trans}(j\omega)^{n_{trans}}}$. Further assignment is also given by assuming that the resistive elements TIF and paracellular pathway both possess simple resistance, R_{TIF} and R_{para} correspondingly. Combining the above definitions give the total impedance of the proposed circuit, Z_{Total} as

$$Z_{Total} = R_{TIF} + Z_{DL} + \left(\frac{1}{R_{para}} + \frac{1}{Z_{trans}}\right)^{-1} \quad (1)$$

$$= R_{TIF} + Z_{DL} + \left[\frac{Z_{trans}R_{para}}{Z_{trans}+R_{para}}\right] \quad (2)$$

Replacing Z_{DL} and Z_{trans} with their corresponding definitions gives the overall circuit impedance as

$$Z_{Total} = R_{TIF} + \frac{1}{A_{DL}(j\omega)^{n_{DL}}} + \frac{\frac{1}{A_{trans}(j\omega)^{n_{trans}}}R_{para}}{\frac{1}{A_{trans}(j\omega)^{n_{trans}}}+R_{para}} \quad (3)$$

The proposed equivalent circuit model was used to fit the obtained impedance spectra measured using IMOD equipped with copper – copper electrodes within subcutaneous E0771 tumor (Supplementary Fig. 18b). A representative impedance spectrum was also given for impedance recorded using implanted IMOD with platinum – copper electrodes under similar experimental settings (Supplementary Fig. 18c). Fitting results were calculated and plotted using Echem Analyst™ software by Gamry Instruments. The parameters for this equivalent circuit model are listed in Supplementary Table 1 with mean values and standard deviations.

Due to the fact that IMOD was inserted into solid tumor when performing the electrochemical impedance spectroscopy, the effective thickness or the distance of the TIF that contributes to the R_{TIF} is relatively small. Moreover, since the ionic composition of TIF is very close to that of plasma, which has the electrical conductivity of 14.3 mS cm^{-1} at body

temperature,[13, 14] we hypothesize that R_{TIF} is negligible in the circuit which is further validated by the equivalent circuit fitting results shown in Supplementary Table 1.

From the averaged fitted results of the E0771 tumors, we observe that the paracellular resistance and transcellular impedance are the critical components in the impedance composition. Next, we investigated the relations between the normalized tumor size and paracellular resistance (R_{para}) and transcellular impedance ($1/A_{trans}$, as the impedance of a capacitor is inversely proportional to its capacitance). As shown in Supplementary Fig. 19, we discovered that the normalized paracellular resistance and transcellular impedance increased linearly with the normalized tumor size, which correlated to our findings of the relationship between the normalized impedance value at a frequency of 10 kHz and the tumor size (Fig. 2). This phenomenon can be further explained by the increase in number of tumor cells as tumor gets larger, leading to the proportional increase of the R_{para} and $1/A_{trans}$, which results in the proportional increase of the total impedance Z_{TOTAL}. This linear relationship between the normalized tumor size and normalized impedance may potentially indicate that the growth pattern of the tumor could be associated with the increase of the impedance at a constant rate.

5. References

1. Kricheldorf HR, Kreiser-Saunders I, Boettcher C. Polylactones: 31. Sn(II)octoate-initiated polymerization of L-lactide: a mechanistic study. *Polymer* **36**, 1253-1259 (1995).
2. Francis DM, *et al.* Blockade of immune checkpoints in lymph nodes through locoregional delivery augments cancer immunotherapy. *Sci. Transl. Med.* **12**, eaay3575 (2020).
3. DiLillo DJ, Yanaba K, Tedder TF. B Cells Are Required for Optimal CD4$^+$ and CD8$^+$ T Cell Tumor Immunity: Therapeutic B Cell Depletion Enhances B16 Melanoma Growth in Mice. .*J. Immunol.* **184**, 4006-4016 (2010).
4. Li Q, *et al.* Adoptive Transfer of Tumor Reactive B Cells Confers Host T-Cell Immunity and Tumor Regression. *Clin. Cancer Res.* **17**, 4987-4995 (2011).
5. Wei SC, *et al.* Distinct Cellular Mechanisms Underlie Anti-CTLA-4 and Anti-PD-1 Checkpoint Blockade. *Cell* **170**, 1120-1133.e1117 (2017).
6. Wei SC, Duffy CR, Allison JP. Fundamental Mechanisms of Immune Checkpoint Blockade Therapy. *Cancer Discov.* **8**, 1069-1086 (2018).
7. Curran MA, Montalvo W, Yagita H, Allison JP. PD-1 and CTLA-4 combination blockade expands infiltrating T cells and reduces regulatory T and myeloid cells within B16 melanoma tumors. *Proc. Natl. Acad. Sci. U. S. A.* **107**, 4275-4280 (2010).
8. Miller BC, *et al.* Subsets of exhausted CD8+ T cells differentially mediate tumor control and respond to checkpoint blockade. *Nat. Immunol.* **20**, 326-336 (2019).
9. Benson K, Cramer S, Galla HJ. Impedance-based cell monitoring: barrier properties and beyond. *Fluids Barriers CNS* **10**, 5 (2013).
10. Venkatanarayanan A, Keyes TE, Forster RJ. Label-free impedance detection of cancer cells. *Anal. Chem.* **85**, 2216-2222 (2013).
11. Stanica L, *et al.* Electric Cell-Substrate Impedance Sensing of Cellular Effects under Hypoxic Conditions and Carbonic Anhydrase Inhibition. *J. Sensors* **2017**, 9290478 (2017).
12. Amini M, Hisdal J, Kalvøy H. Applications of bioimpedance measurement techniques in tissue engineering. *J. Electr. Bioimpedance* **9**, 142-158 (2018).
13. Duck FA. Chapter 6 - Electrical Properties of Tissue. In: *Physical Properties of Tissues* (eds Duck FA). Academic Press (1990).
14. Wagner M, Wiig H. Tumor Interstitial Fluid Formation, Characterization, and Clinical Implications. *Front. Oncol.* **5**, 115 (2015).
